# Parental diabetes and birthweight in 236 030 individuals in the UK Biobank Study

**DOI:** 10.1093/ije/dyt220

**Published:** 2013-12-10

**Authors:** Jessica S Tyrrell, Hanieh Yaghootkar, Rachel M Freathy, Andrew T Hattersley, Timothy M Frayling

**Affiliations:** ^1^European Centre for Environment and Human Health, University of Exeter Medical School, Truro, UK, ^2^Genetics of Complex Traits, University of Exeter Medical School, Exeter, UK and ^3^Molecular Genetics, Wonford Building, University of Exeter Medical School, Exeter, UK

**Keywords:** Type 2 diabetes, parental history, birthweight, UK Biobank, genetics

## Abstract

**Background** The UK Biobank study provides a unique opportunity to study the causes and consequences of disease. We aimed to use the UK Biobank data to study the well-established, but poorly understood, association between low birthweight and type 2 diabetes.

**Methods** We used logistic regression to calculate the odds ratio for participants’ risk of type 2 diabetes given a one standard deviation increase in birthweight. To test for an association between parental diabetes and birthweight, we performed linear regression of self-reported parental diabetes status against birthweight. We performed path and mediation analyses to test the hypothesis that birthweight partly mediates the association between parental diabetes and participant type 2 diabetes status.

**Results** Of the UK Biobank participants, 277 261 reported their birthweight. Of 257 715 individuals of White ethnicity and singleton pregnancies, 6576 had type 2 diabetes, 19 478 reported maternal diabetes (but not paternal), 20 057 reported paternal diabetes (but not maternal) and 2754 participants reported both parents as having diabetes. Lower birthweight was associated with type 2 diabetes in the UK Biobank participants. A one kilogram increase in birthweight was associated with a lower risk of type 2 diabetes (odds ratio: 0.74; 95% CI: 0.71, 0.76; *P* = 2 × 10^−57^). Paternal diabetes was associated with lower birthweight (45 g lower; 95% CI: 36, 54; *P* = 2 × 10^−23^) relative to individuals with no parental diabetes. Maternal diabetes was associated with higher birthweight (59 g increase; 95% CI: 50, 68; *P* = 3 × 10^−37^). Participants’ lower birthweight was a mediator of the association between reported paternal diabetes and participants’ type 2 diabetes status, explaining 1.1% of the association, and participants’ higher birthweight was a mediator of the association between reported maternal diabetes and participants’ type 2 diabetes status, explaining 1.2% of the association.

**Conclusions** Data from the UK Biobank provides the strongest evidence by far that paternal diabetes is associated with lower birthweight, whereas maternal diabetes is associated with increased birthweight. Our findings with paternal diabetes are consistent with a role for the same genetic factors influencing foetal growth and type 2 diabetes.

## Introduction

The UK Biobank study provides a unique opportunity to study the causes and consequences of disease. It consists of approximately 500 000 UK adults with extensive baseline data. Of these individuals, 99.5% were aged between 40 and 70 years at the time of study. The main aim is to study risk factors, including gene-environment interactions, associated with incident disease, but the size of the study provides opportunities to further the understanding of disease using prevalent cases. In this study we aimed to use the UK Biobank data to study the well-established, but poorly understood, association between low birthweight and type 2 diabetes.

Both genetic and environmental factors are possible explanations for the association between low birthweight and higher risk of type 2 diabetes.^1^^,^^2^ If the same genetic factors contribute to low birthweight and diabetes risk, then interventions that target the *in utero* environment to increase birthweight are less likely to alter the risk of adult disease. There is good evidence from human studies that the *in utero* environment can programme foetal metabolism, but this evidence comes mostly from the associations between an adverse *in utero* environment and higher birthweight. For example, exposure to maternal diabetes (types 1 and 2) *in utero* is associated with higher birthweight and adverse outcomes including offspring diabetes risk and adiposity, most likely as a result of higher glucose levels.^3–6^ The evidence for an *in utero* programming effect of lower birthweight is more limited and includes studies of exposure to famine *in utero* and data from twin studies. Exposure of pregnant women to famine during the Dutch ‘Hunger Winter’ was associated with impaired glucose tolerance in their adult offspring,^7^ although statistical robustness and replication of these data are limited.^8^ Discordant monozygotic twin studies have supported a non-genetic basis for the association between birthweight and adult onset disease.^9^

The foetal insulin hypothesis^2^ proposed that common genetic variants that influence insulin secretion or action may play a role in both the reduction of foetal growth and the increased risk of type 2 diabetes and related diseases in adulthood. The hypothesis was initially based on observations in monogenic diabetes, but subsequent evidence has supported the foetal insulin hypothesis in type 2 diabetes. Strong support came from the finding that 660 Pima Indian individuals with a paternal history of diabetes were lighter at birth compared with 948 individuals with no parental history of diabetes.^3^ Evidence for or against a relationship between parental type 2 diabetes and birthweight is very weak in European populations because the prevalence of type 2 diabetes is low and typical studies have only been able to analyse 350 cases at most of parental diabetes.^10^^,^^11^ A recent study demonstrated that men who fathered growth-restricted offspring have preclinical evidence of insulin resistance syndrome.^12^ Further evidence suggests that low birthweight is associated with increased paternal blood pressure, glucose, lipids and body mass index.^13^ Recent genome-wide association studies have provided some evidence that a genetic link exists. These studies identified common genetic variants where the same allele of the same variant is associated with both a higher risk of type 2 diabetes and lower birthweight.^14^^,^^15^

In this study we used the UK Biobank study of approximately 500 000 individuals, including 257 715 White individuals with self-reported birthweight available (excluding multiple births), 6576 of whom we defined as having type 2 diabetes, and 42 289 of whom had a reported history of parental diabetes. We used these data to estimate the association between participants’ birthweight and type 2 diabetes and to provide estimates of the associations between reported paternal or maternal diabetes and participants’ birthweight. We had two related hypotheses. First, that participants’ birthweight would be associated with type 2 diabetes; and second, that paternal diabetes would be associated with lower birthweight, providing evidence that genetic factors contribute to the association between low birthweight and type 2 diabetes.

## Methods

### Participants

The UK Biobank recruited 502 713 people aged 37–73 years (99.5% were between 40 and 69 years) in 2006–10 from across the country. Participants provided a large amount of information about themselves via questionnaires (including information about demographics, health status, early life, diet and lifestyle) and anthropometric measurements, blood, urine and saliva samples were taken for future analysis: this has been described in more detail elsewhere.^16^ To investigate associations in individuals of European descent we included data on the 472 883 White individuals.

### Testing the validity of self-reported birthweight

**Figure 1:**
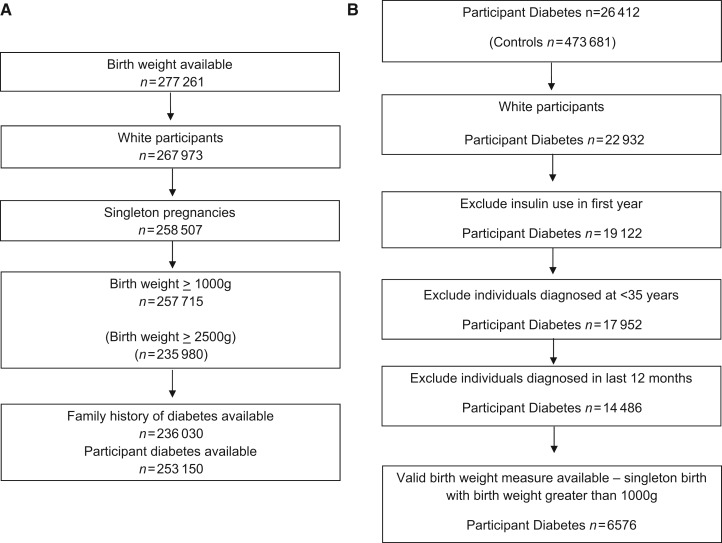
Flow chart showing how we defined (i) valid birthweight measures and (ii) participant type 2 diabetes in the UK Biobank

All participants in the UK Biobank were asked by the nurse conducting the interview to report their birthweight. Participants were first asked ‘Do you know your birthweight?’ with the option to select yes in pounds and ounces, yes in kilograms or no. They were then asked to input their birthweight in the format they could remember. All birthweights were then converted into kilograms. As the UK Biobank only includes self-reported birthweight (rather than hospital records), it is important to assess the validity of these data by examining the usually observed associations. Self-reported birthweight was available for 267 973 White participants (7442 from multiple pregnancies; Figure 1a). To assess the validity of self-reported birthweight, we tested the associations between birthweight and non-singleton pregnancies, female sex, self-reported maternal smoking at the time of pregnancy, earlier year of birth and socioeconomic status (Table 1). For analyses with diabetes status and parental diabetes status, we excluded the 7442 participants who reported being part of a multiple birth, the 2024 individuals who failed to report whether they were part of a multiple birth and the 792 individuals who reported weighing <1000 g at birth (Figure 1a). No other information indicating prematurity or gestational age was available.

**Table 1:** Characteristics of the 257 715 White UK Biobank participants from singleton pregnancies with birthweight data available, where birthweight is ≥1000 g. The mean birthweight has also been summarized for each characteristic investigated. *P*-values represent the difference in birthweight for different characteristics. Note the multiple births data include 7185 additional individuals reported as part of a multiple birth

		Number (%)	Mean birthweight g (standard deviation)	*P*
**Mean age at recruitment, years (SD)**	55.33 (8.09)		3358 (632)	
**Year of birth**	1934–1943	37 176 (14.43)	3344 (711)	<0.0001
	1944–1953	104 400 (40.51)	3362 (646)	
	1954–1963	83 671 (32.47)	3353 (596)	
	1964–1971	32 468 (12.60)	3371 (577)	
**Sex**	Male	100 439 (38.97)	3484 (650)	<0.0001
	Female	157 276 (61.03)	3277 (607)	
**Ethnicity**	White	234 (0.09)	3402 (747)	<0.0001
	White British	242 987 (94.29)	3356 (630)	
	White Irish	5995 (2.33)	3411 (692)	
	White other	8499 (3.30)	3362 (652)	
**Multiple births**	Yes	7185 (2.71)	2489 (690)	<0.0001
	No	257 715 (97.21)	3358 (632)	
**Maternal smoking**	Yes	68 332 (26.51)	3288 (659)	<0.0001
	No	162 889 (63.21)	3387 (614)	
	Unknown	26 468 (10.27)	3355 (659)	
	Prefer not to answer	16 (0.01)	3356 (637)	
**Townsend deprivation index**	−6.26−(–)3.64	68 671 (26.65)	3366 (614)	<0.0001
	−3.64−(–)2.14	66 961 (25.98)	3361 (625)	
	−2.13–0.55	65 004 (25.22)	3356 (632)	
	0.55–11.00	56 739 (22.02)	3345 (663)	
	Missing	340 (0.13)	3398 (615)	
**Participant type 2 diabetes**	No	246 574 (95.79)	3361 (628)	<0.0001
	Yes	6576 (2.55)	3274 (734)	
	Unknown	4269 (1.66)	3303 (696)	
**Parental diabetes**	Neither	193 741 (75.20)	3358 (622)	<0.0001
	Father only	20 057 (7.78)	3311 (602)	
	Mother only	19 478 (7.56)	3415 (675)	
	Both	2754 (1.07)	3370 (662)	
	Missing	21 685 (8.41)	3348 (696)	

### Participant diabetes

We defined 14 486 White participants as having type 2 diabetes (Figure 1b) (6576 of whom also reported their birthweight). Participants were defined as having type 2 diabetes if they had answered yes to the question ‘Has a doctor ever told you that you have diabetes?’ and had not reported using insulin within the first year of diagnosis. We excluded individuals who reported that they were diagnosed under the age of 35 years (*n* = 682), to limit the numbers of individuals with slow-progressing autoimmune diabetes or monogenic forms of diabetes. We also excluded individuals reporting diabetes diagnosed within the past year (*n* = 1757) to exclude those who may be given insulin within the first year of diagnosis, therefore suggesting they may have a slow onset form of type 1 diabetes.

### Parental diabetes

To investigate parental disease, participants were asked ‘Has/did your father ever suffer from heart disease, stroke, high blood pressure, diabetes, chronic bronchitis, Alzheimer’s disease?’ and, if they answered yes, participants were asked to select the appropriate disease(s) from a list. An identical question was asked about participants’ mothers. Participants reporting that they did not know about parental conditions or who preferred not to answer were excluded from this analysis (*n* = 31 055 for paternal conditions and *n* 18 481 for maternal conditions). These participants were very slightly smaller at birth compared with those who did report their parents’ disease status after correcting for socio-economic status (3379 g versus 3398 g; *P* = 0.04). It was not possible to distinguish between type 1 and type 2 parental diabetes, but type 1 is relatively rare compared with type 2 diabetes (type 2 diabetes accounts for 85% of diabetes cases in the UK^5^).

### Association analyses

#### Parental diabetes status and participant diabetes status

We investigated the association between parental diabetes status and participant diabetes status using logistic regression models. We calculated odds ratios and 95% confidence intervals (OR; 95% CI) for risk of type 2 diabetes if (i) either parent had diabetes, (ii) paternal diabetes was reported, (iii) maternal diabetes was reported and (iv) both parents reported to have diabetes. We used age and sex as covariates.

#### Participants’ birthweight compared with type 2 diabetes status

We created birthweight quintiles for all participants to investigate the association between birthweight and an individual’s subsequent risk of developing type 2 diabetes (quintile 1: mean 2.44 kg (standard deviation 0·40 kg); quintile 2: 3.08 kg (0.11 kg); quintile 3: 3.33 kg (0.07 kg); quintile 4: 3.62 kg (0.09 kg); and quintile 5: 4.24 (0.40 kg)). We calculated odds ratios and 95% confidence intervals (OR; 95% CI) for risk of type 2 diabetes in each birthweight quintile compared with quintile 1. We also calculated the odds ratio for type 2 diabetes given one standard deviation change in birthweight. We used sex (as male babies were approximately 200 g heavier than females in the UK Biobank data), year of birth (to account for secular increases in birthweight), self-reported maternal smoking around the time of birth, participant Townsend deprivation index and participant home location at birth as covariates. We also calculated sex-specific birthweight quintiles for the UK Biobank participants and investigated the association between birthweight and an individual’s subsequent type 2 diabetes risk in males and females separately.

#### Parental diabetes status compared with participants’ birthweight

To test for an association between parental diabetes and birthweight, we performed linear regression of self-reported parental diabetes status as the independent variable (coded as no parental history of diabetes, paternal diabetes only, maternal diabetes only or both parents diabetic) and birthweight as the dependent variable, with participant sex, year of birth, self-reported maternal smoking around the time of birth, participant Townsend deprivation index and participant home location at birth as covariates.

### Path and mediation analyses

Path analysis, a special case of structural equation modelling (SEM) wherein all variables are observed and no latent variables are estimated, was used to test the interdependent relationship between parental diabetes, participant diabetes and birthweight. Model goodness of fit was evaluated using the chi-square test and three fit indices, namely the comparative fit index (CFI), root mean square error of approximation (RMSEA) and the standardized root mean square residual (SRMR). Acceptable fit was defined as: CFI >0.95, RMSEA <0.06 and SRMR <0.08.

We also performed a bootstrapped mediation analysis using the user-written binary mediation command in STATA as we were utilizing two binary variables. We used this mediation analysis to investigate whether or not participant’s birthweight mediated associations between each parent’s reported diabetes status and participant’s type 2 diabetes status.

### Sensitivity analyses

We performed a sensitivity analysis where all individuals reporting a birthweight of <2500 g were excluded from the analyses (*n* = 21 735). This was to ensure that included individuals were unlikely to be preterm births.^17^ We also performed a sensitivity analysis including the 1757 participants who were diagnosed with diabetes within the year prior to recruitment to the UK Biobank.

All analyses were conducted using STATA/IC Version 12.1 (College Station, TX).

## Results

### Validity of self-reported birthweight

Self-reported birthweight was available for 277 261 participants (257 715 singletons) and was associated with non-singleton pregnancies, female sex, self-reported maternal smoking at the time of pregnancy, earlier year of birth and socioeconomic status (Table 1) in the expected directions.

### The association between parental diabetes and participant diabetes status

Participants reporting a parental history of diabetes had an increased risk of type 2 diabetes (OR 3.65; 95% CI 3.52, 3.79). This association was observed when paternal history only (3.01; 2.85, 3.18), maternal history only (3.69; 3.52, 3.88) or when both paternal and maternal diabetes were reported (8.60; 7.78, 9.53).

### The association between participant birthweight and their diabetes status

**Figure 2:**
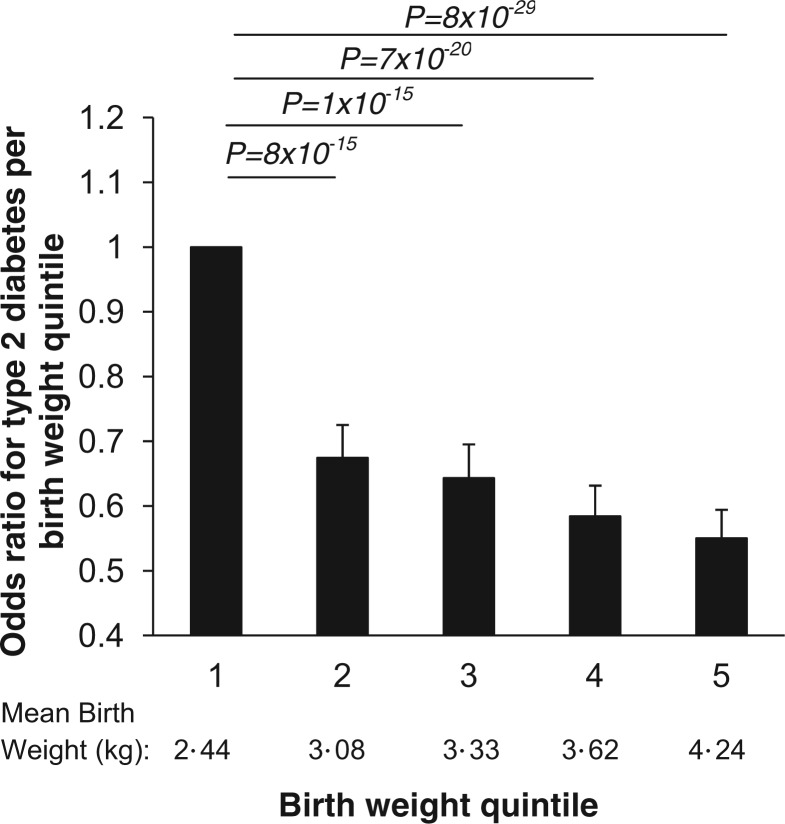
Bar chart presenting the odds ratios for type 2 diabetes by birthweight quintile in a basic sex- and year of birth-adjusted analyses; odds ratios presented are relative to the lowest birthweight quintile (OR 1). Absolute *P-*values for each quintile compared with other quintiles are presented for the fully adjusted model. Error bars represent 95% confidence intervals

The characteristics of the 257 715 White UK Biobank individuals from singleton births with birthweight data are summarized in Table 1. Of the 257 715 White UK Biobank individuals with birthweight data available, 6576 were defined as having type 2 diabetes. Higher birthweight was associated with lower odds of type 2 diabetes later in life. The risk of type 2 diabetes decreased in groups of individuals of higher birthweight (Figure 2); compared with quintile 1: quintile 2 OR 0.67; 95% CI 0.63, 0.73, quintile 3 OR 0.64; 0.59, 0.70, quintile 4 OR 0.58; 0.54, 0.63, quintile 5 OR 0.55; 0.51, 0.59; *P* <0.0001 for trend across groups). Additional adjustment for self-reported maternal smoking, Townsend deprivation index and participant place of birth resulted in a similar inverse relationship between birthweight and type 2 diabetes risk (Supplementary Figure 1a, available as Supplementary data at *IJE* online). Sex-specific quintiles produced similar results with higher birthweight quintiles associated with lower type 2 diabetes risk in later life (Supplementary Figure 1b, available as Supplementary data at *IJE* online).

Inclusion of the 1757 individuals reporting a diagnosis of diabetes within the last 12 months did not alter our findings (quintile 2 OR 0.68; 0.64, 0.73, quintile 3 OR 0.66; 0.61, 0.70, quintile 4 0.61; 0.57, 0.65], quintile 5 0.56; 0.52, 0.60; *P* < 0.0001 for trend). Associations were also similar when only individuals with a birthweight of 2500 g or more were included (quintile 2 OR 0.72; 0.64, 0.82], quintile 3 OR 0.75; 0.67, 0.86], quintile 4 0.70; 0.62, 0.80], quintile 5 0.70; 0.61, 0.79; *P* < 0.0001 for trend).

### The association between participant birthweight and parental diabetes status

**Figure 3:**
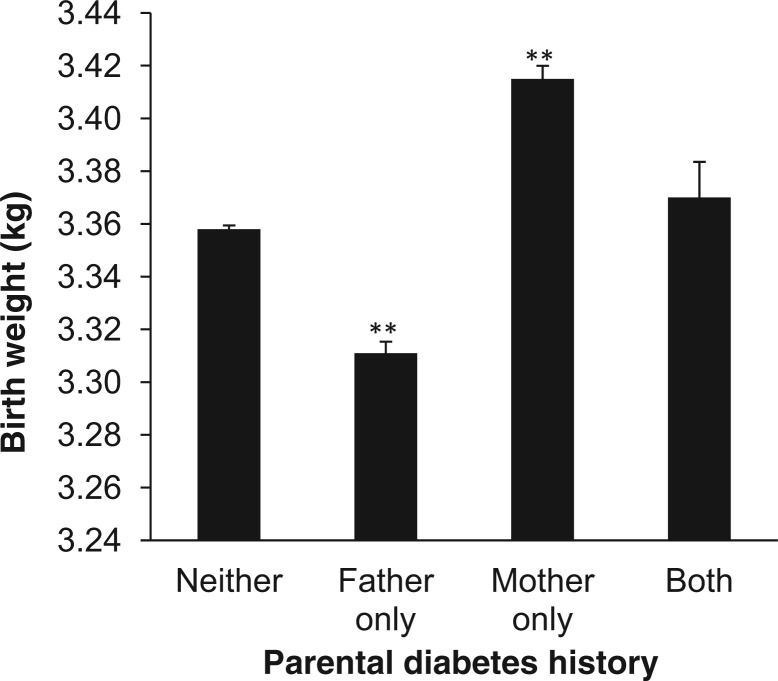
Bar chart representing the mean birthweight of individuals classified in terms of their family history of type 2 diabetes. **P < 0·001 in birthweight associations when individuals with a reported parental history of diabetes are compared with those with neither parent reported as diabetic

Parental diabetes status was associated with birthweight (Figure 3 and Supplementary Figure 2, available as Supplementary data at *IJE* online; overall difference between groups *P* < 0.0001). The 19 478 individuals reporting a history of maternal diabetes (but no paternal history) were heavier at birth (0.059 kg; 95% CI 0.050, 0,068) (Table 2) when compared with participants with no family history of diabetes. Similar effect sizes were noted when maternal diabetes was compared with all others regardless of whether or not the participant had reported a history of paternal diabetes (0.057 kg; 95% CI 0.049, 0.065). The 20 057 individuals reporting a history of paternal diabetes (but no maternal history) were lighter at birth (−0.045 kg; 95% CI −0.054, −0.036). When participants reporting paternal diabetes were compared against all others, similar effect sizes were noted (−0.044 kg; 95% CI −0.052, −0.035). For the 2754 participants reporting both parents as diabetic, there was no association with birthweight (0.009 kg; 95% CI −0.016, 0.033) when compared with participants reporting no parental diabetes. The effect size estimates observed for both paternal and maternal diabetes were similar when adjusting for maternal smoking around the time of pregnancy, the Townsend deprivation index and location of home at birth (Table 2) and when excluding the 21 735 participants with a birthweight <2500 grams (Supplementary Table 1, available as Supplementary data at *IJE* online).

**Table 2:** Association between parental history of diabetes and birthweight

Sample group	Analysis details	Total *n* of parental diabetes (controls)	Difference in birthweight in kilograms relative to no fa mily history of diabetes (95% CI)	Association *P*-value
Paternal diabetes only	Basic analysis^a^	20 057 (193 741)	–0·045 (–0·054, −0·036)	2 × 10^−23^
	Maternal smoking around pregnancy and SES included as covariables	15 467 (149 991)	–0·043 (–0·053, −0·034)	1 × 10^−18^
Maternal diabetes only	Basic analysis^a^	19 478 (193 741)	0·059 (0·050, 0·068)	3 × 10^−37^
	Maternal smoking around pregnancy and SES included as covariables	15 038 (149 991)	0·061 (0·052, 0·072)	8 × 10^−35^
Both maternal and paternal diabetes	Basic analysis^a^	2754 (193 741)	0·009 (-0·013, 0·033)	0·43
	Maternal smoking around pregnancy and SES included as covariables	2140 (149 991)	0·005 (-0·020, 0·029)	0·69

SES, socio-economic status as measured by the Townsend deprivation index at recruitment and home location (based on north and east coordinates) at birth.

^a^Basic analysis: linear regression of reported parental diabetes status (coded as 0 (no family history) or 1 (mother and/or father with type 2 diabetes) against birthweight, with sex and year of birth as covariables.

### Path analysis and mediation analysis

In Figure 3 we show the unstandardized path coefficients for the fitted parent–participant diabetes model. As the model was saturated, overall fit was perfect (chi-square = 0; CFI = 1.0; RMSEA = 0; SRMR = 0). This model details the associations when the four variables are considered in one model (Figure 3). We observed similar associations as previously reported. Both paternal and maternal diabetes were positively associated with participant diabetes. Maternal diabetes was associated with higher birthweight (60 g; 95% CI 51, 68), and paternal diabetes was associated with lower birthweight (−44 g; 95% CI −53, −36). Birthweight was inversely associated with participant diabetes.

**Figure 4:**
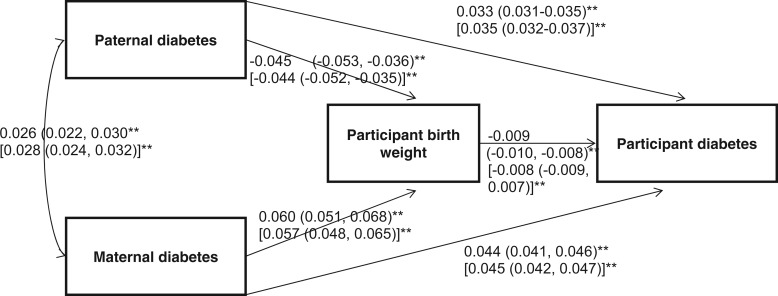
Path analysis of associations between participant birthweight, parental history of diabetes and participant diabetes. **P < 0.001. The regression coefficients presented in brackets represent the coefficients achieved when single pairwise associations are investigated

We also performed a mediation analysis to determine if birthweight was a mediator in the parental diabetes, participant type 2 diabetes associations. We separated these analyses by parental sex. Participants’ lower birthweight was a partial mediator of the association between reported paternal diabetes and participants’ type 2 diabetes status and explained 1.1% of the association (indirect effect via birthweight 0.0016; 0.0011, 0.0020 and direct effect 0.140; 0.130, 0.150; *P* < 0.001 for both; Figure 4A). Participants’ higher birthweight was a mediator of the association between reported maternal diabetes and participants’ type 2 diabetes status and explained 1.2% of the association (indirect effect via birthweight 0.0022; 0.00162, 0.00282 and direct effect 0.182 (0.172, 0.190); *P* < 0.001 for both; Figure 4B).

## Discussion

The ability to study more than one-quarter million UK people with birthweight and parental diabetes data enabled us to provide several advances to the understanding of the relationship between birthweight and type 2 diabetes.

### Paternal diabetes is associated with lower birthweight and maternal diabetes is associated with higher birthweight

First, we provide the strongest evidence by far that paternal diabetes is associated with lower birthweight. The 20 057 UK Biobank individuals with a paternal history of diabetes were 45 g (95% CI 36, 54) lighter at birth compared with individuals with no parental history of diabetes (*P* = 2 × 10^−23^). Our data also provide very strong evidence that mothers at risk of late onset diabetes have heavier babies. Although the association between maternal diabetes and higher birthweight is well established, and the association between paternal diabetes and lower birthweight has been described, the size of our study and the statistical confidence of our results provide the most robust evidence that paternal diabetes is associated with lower birthweight.

**Figure 5:**
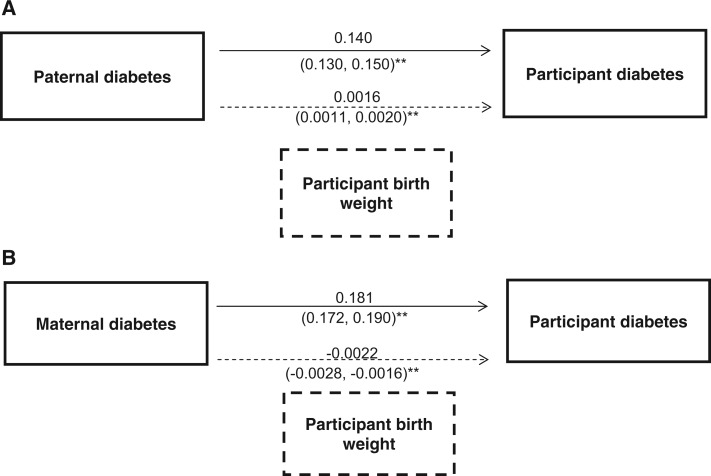
Direct and indirect effects of (A) paternal and (B) maternal diabetes on participant diabetes. The indirect effect (dashed arrow) represents the proportion of the effect mediated by participant birthweight. **P < 0.001

### Lower birthweight is associated with participant’s own risk of type 2 diabetes

Second, the large sample size provided very robust statistical evidence that lower birthweight is associated with type 2 diabetes in adulthood. This adds to the evidence from many studies that have demonstrated an inverse relationship between birthweight and subsequent type 2 diabetes^18^ but does not in itself help us understand the mechanism behind this association.

### Self-reported birthweight is a valid measure of birthweight

A third finding from our study is that self-reported birthweight represents a valid estimate of birthweight. Although likely to be less precise than maternal reports or measured birthweight, lower self-reported birthweight was associated with female sex, increased deprivation index, multiple births and self-reported maternal smoking. These associations were not only in the expected direction, but demonstrated effect sizes which have been demonstrated in other studies with more accurate birthweight data available.^19^^,^^20^ This is in keeping with a recent study of 541 individuals where a positive correlation was observed between self-reported birthweight in the Million Women Study and their recorded birthweight in 1946, as part of the MRC National Survey of Health and Development birth cohort.^21^

### Parental diabetes is associated with increased odds of participant doctor-diagnosed diabetes

We noted robust associations between parental diabetes and increased odds of participant diabetes. The odds ratios reported here were similar to other studies with more accurate diabetes information available.^22^ This suggests that the self-reported data on parental history of diabetes and participant diabetes are valid.

### Birthweight is a partial mediator of the associations between parental diabetes and participants’ type 2 diabetes

Our mediation analysis provides the first evidence that the association between paternal diabetes and participants’ type 2 diabetes status is partly mediated through birthweight. The most likely explanation for this effect is through genetic variation passed from father to child, that influences both lower birthweight and increased risk of type 2 diabetes. Although the birthweight-mediated effect only accounted for 1% of the association between paternal and participant diabetes, this has to be put in the context of the many potential postnatal factors that are likely to cause an association between fathers’ and offsprings’ risk of type 2 diabetes over decades compared with a few months of prenatal life. Our mediation analysis also provides evidence that the association between maternal diabetes and participants’ type 2 diabetes status is partly mediated through birthweight. This effect could be caused by intra-uterine programming or genetics or a combination of these.

### Strengths and limitations of the study

The main strength of our study is sample size. We assessed the relationship between birthweight and parental diabetes status in 236 030 individuals, of whom 42 289 reported a history of parental diabetes. These data compare with previous studies of 1608 Pima Indians of whom 660 had a parental history of diabetes, and 335 Norwegians with diabetes reported as cause of paternal death.

We acknowledge some limitations to our study. First, the disease diagnoses were not gold standard, with parental and personal history being self-reported. For diabetic individuals, especially in parents where we did not know time to insulin treatment, we could not distinguish between type 1 and type 2 diabetes. However, given the age of the parents and the fact that type 2 diabetes is far more prevalent, we anticipate that the majority of cases of reported parental history of diabetes would be type 2. Second, birthweight was self-reported and no gestational age data were available. This limitation meant that, although much larger than previous studies addressing the same question, birthweight was not measured as accurately in UK Biobank compared with smaller previous studies. However, we observed expected associations with well-known covariates including sex, maternal smoking and year of birth. Third, we could not account for factors such as non-paternity, but this would have biased data to false negative rather than false positive findings.

### Interpretation

Our study provides evidence that genetic factors contribute to the association between reduced intrauterine growth and diabetes. If this is the major factor, it would cast doubt on the likely success of interventions aimed at reducing the burden of diabetes through improving *in utero* growth. It is difficult to compare the effect of paternal diabetes, likely reflecting genetics, with the effects of intrauterine programming because it is very hard to study the role of maternal undernutrition in humans and studies come from different time periods and populations. However, the effect sizes we observed between paternal diabetes and lower birthweight are approximately one-fifth of those observed when pregnant mothers consumed fewer than 1000 calories a day in late gestation during the Dutch Hunger Winter of 1945.^15^

We believe the association between maternal diabetes, higher birthweight and subsequent participant diabetes in the UK Biobank is likely to be due to a mechanism whereby raised glucose levels in the normal range during pregnancy are associated with both higher birthweight (as previously reported in many studies^23^) and future maternal risk of diabetes. Maternal obesity may contribute to raised glucose in pregnancy and raised birthweight as well as increasing risk of maternal diabetes, but maternal BMI data were not available to test this directly. We do not have age of diagnosis of diabetes in mothers, but the majority will have been diagnosed in later life, long after childbearing age.

In conclusion, our analysis of the UK Biobank data has provided robust evidence of the association between participant-reported parental diabetes status and birthweight. We observed associations between maternal diabetes and increased offspring birthweight and subsequent risk of offspring diabetes. Our study provides the most robust evidence to date that paternal diabetes is associated with lower birthweight in a European population and that the association between paternal diabetes and participants’ type 2 diabetes is partly mediated through intrauterine, most likely genetic, effects.

## Supplementary Data

Supplementary data are available at *IJE* online.

## Funding

This work was supported by investment from the ERDF (European Regional Development Fund) and ESF (European Social Fund) Convergence Programme for Cornwall and the Isles of Scilly (J.S.T.). The Wellcome Trust (WT 085541/Z/08/Z) supports. R.M.F. The European Research Council, Wellcome Trust and Diabetes UK support the research by T.M.F.

## Supplementary Material

Supplementary Data
