# Supplementary material for: Parental diabetes and birthweight in 236 030 individuals in the UK Biobank Study
Source: Int J Epidemiol. 2013 Dec 10;42(6):1714–23. doi: 10.1093/ije/dyt220 (PMC3887570; doi:10.1093/ije/dyt220)
Supplement: Supplementary Data [file supp_42_6_1714__index.html]

Parental diabetes and birthweight in 236 030 individuals in the UK Biobank Study — Parental diabetes and birthweight in 236 030 individuals in the UK Biobank Study — Supplementary Data 

# Parental diabetes and birthweight in 236 030 individuals in the UK Biobank Study

## Supplementary Data

files

**Files in this Data Supplement:**

- Supplementary Data - docx file
