# Supplementary material for: Parental diabetes and birthweight in 236 030 individuals in the UK Biobank Study
Source: Int J Epidemiol. 2013 Dec 10;42(6):1714–23. doi: 10.1093/ije/dyt220 (PMC3887570; doi:10.1093/ije/dyt220)
Supplement: Supplementary Data [file supp_dyt220_ije-2013-03-0334-File002.docx]

A

B

Supplementary Figure 1: Bar chart presenting the odds ratios for type 2 diabetes by birth weight quintile: A) additionally adjusted for Townsend deprivation index (SES) and maternal smoking status (white bars); B) for males (dark grey bars) and females (light grey bars) Odds ratios presented are relative to the lowest birth weight quintile (OR: 1). Error bars represent 95% confidence intervals.

**

**

Figure 2: Bar chart representing the mean birth weight of individuals classified in terms of their family history of type 2 diabetes. The means are adjusted for year of birth, sex, maternal smoking and the Townsend deprivation index. The error bars represent the 95% confidence intervals of the data. ** represent associations at *P*<0·001 in birth weight when individuals with a reported parental history of diabetes are compared to those with neither parent reported as diabetic.

| **Supplementary Table 2.** Association between parental history of diabetes and birth weight where birth weight is greater than or equal to 2500 grams | | | | |
| --- | --- | --- | --- | --- |
| Sample Group | Analysis details | Total *n* of parental diabetes  (controls) | Difference in birth weight in kilograms relative to no family history of diabetes (95%CI) | Association *P*  value |
| Paternal diabetes only | Basic analysis^a^ | 18,291  (177,887) | -0·038 (-0·046, -0·030) | 7x10^-22^ |
|  | Maternal smoking around pregnancy and SES included as covariables | 16,565  (160,701) | -0·037 (-0·045, -0·029) | 8x10^-19^ |
| Maternal diabetes only | Basic analysis^a^ | 17,878  (177,887) | 0·066 (0·059, 0·075) | 9x10^-61^ |
|  | Maternal smoking around pregnancy and SES included as covariables | 16,064  (160,701) | 0·066 (0·057, 0·074) | 3x10^-54^ |
| Both maternal and paternal diabetes | Basic analysis^a^ | 2,526  (177,887) | 0·018 (-0·002, 0·038) | 0.082 |
|  | Maternal smoking around pregnancy and SES included as covariables | 2,281  (160,701) | 0·018 (-0·002, 0·039) | 0.090 |
